# Chlamydia screening in England: a qualitative study of the narrative behind the policy

**DOI:** 10.1186/1471-2458-12-317

**Published:** 2012-04-30

**Authors:** Jessica Sheringham, Paula Baraitser, Ian Simms, Graham Hart, Rosalind Raine

**Affiliations:** 1 UCL Department of Applied Health Research, Department of Epidemiology and Public Health, UCL, 1-19 Torrington Place, London, WC1E 6BT, UK; 2 National Chlamydia Screening Programme, Health Protection Services, Health Protection Agency, 61 Colindale Avenue, London, NW9 5EQ, UK; 3 Kings College Hospital NHS Foundation Trust, Camberwell Sexual Health Centre, 100 Denmark Hill, London, SE5 9RS, UK; 4 HIV & STI Department, Health Protection Services, Colindale, Health Protection Agency, 61 Colindale Avenue, London, NW9 5EQ, UK; 5 Faculty of Population Health Sciences, School of Life & Medical Sciences, University College London, London, WC1E 6BT, UK; 6 UCL Partners Programme Director for Population Health, UCL Centre of Applied Health Research, Department of Epidemiology and Public Health, UCL, 1-19 Torrington Place, London, WC1E 6BT, UK

## Abstract

**Background:**

The rationale for the English National Chlamydia Screening Programme (NCSP) has been questioned. There has been little analysis, however, of what drove the NCSP’s establishment and how it was implemented. Such analysis will help inform the future development of the NCSP. This study used a qualitative, theory-driven approach to evaluate the rationale for the NCSP’s establishment and implementation.

**Methods:**

Semi-structured interviews with 14 experts in chlamydia screening were undertaken. The interview data were analysed with policy documents and commentaries from peer-reviewed journals (published 1996–2010) using the Framework approach.

**Results:**

Two themes drove the NCSP’s establishment and implementation. The first, chlamydia control, was prominently referenced in documents and interviews. The second theme concerned the potential for chlamydia screening to advance wider improvements in sexual health. In particular, screening was expected to promote sexual health services in primary care and encourage discussion of sexual health with young people. While this theme was only indirectly referenced in policy documents, it was cited by interviewees as a strong influence on implementation in the early years. However, by full rollout of the Programme, a focus on screening volume may have limited the NCSP’s capacity to improve broader aspects of sexual health.

**Conclusions:**

A combination of explicit and implicit drivers underpinned the Programme’s establishment. This combination may explain why there was widespread support for its introduction and why implementation of the NCSP was inconsistent. The potential to improve young people’s sexual health more comprehensively should be made explicit in future planning of the NCSP.

## Background

The National Chlamydia Screening Programme (NCSP) was established in England in 2002 to “prevent and control chlamydia through early detection and treatment of asymptomatic infection; reduce onward transmission to sexual partners; and prevent the consequences of untreated infection”. It has been available in all areas of England since 2008 and in 2009/2010 provided over 1.5 million tests to young people [1].

When chlamydia screening was proposed in 1998 by an Expert Advisory Group of the Chief Medical Officer, it was expected to “produce considerable health gains” and “reduce health costs” by preventing reproductive ill health (eg pelvic inflammatory disease, ectopic pregnancy and tubal factor subfertility) thought to be caused by chlamydia infection [2]. Eleven years later, the National Audit Office questioned whether it was worth investing “so much public money” to tackle chlamydia when the evidence base for screening was “subject to debate” [3]. While this and other reports have criticised the NCSP [4], there has been little objective analysis of the broader factors driving the Programme’s establishment or exploration of decisions underlying its implementation. Such analysis is needed to inform decisions about the NCSP’s future. It is important at this time when extensive healthcare reforms underway across the English National Health Service will challenge the NCSP’s management and direction [5].

The NCSP is not unique in attracting controversy or in being established on the basis of limited evidence: for example, historical analysis of the cervical screening programme illustrates a similarly optimistic initial response, despite scant evidence, and later concerns about a lack of evidence of benefit [6]. Indeed, new interventions are often informed by plausible intuition rather than evidence-based pathways describing anticipated outcomes [7]. In some instances, such approaches are grounded in theory but in many cases they are based on implicit assumptions. It is helpful to distinguish between these approaches in order to fully understand the motivations for the establishment of new interventions and to explain the effectiveness of widespread adoption and dissemination of the intervention.

### Aims and objectives

The aim of this study was to explore implicit and explicit determinants of the establishment and implementation of the English National Chlamydia Screening Programme to inform the future of chlamydia control and sexual health programmes for young people. The objectives were to:

Track the scientific and policy context under which the NCSP was first considered, established and implemented;

Make explicit any initial theory and assumptions underlying the policy to introduce a national chlamydia screening programme in England;

Document changes in, and challenges to, these drivers as the NCSP was implemented.

## Methods

### Approach

Theory-driven evaluation (TDE) encompasses a family of approaches that first seeks to articulate the links between what programme designers planned to do and what they expected to attain. This requires making implicit assumptions explicit so they can be subject to critical appraisal and identifying the full spectrum of possible impacts of the intervention [8,9]. TDE then goes on to explain programme outcomes in the light of any underlying theory identified, other assumptions or motivating factors. This first stage of understanding the intervention requires a qualitative approach. TDE acknowledges that interpretation of the underlying theory or other drivers for the programme may change as the programme is rolled out and is subject to further scrutiny [10]. A recent TDE of a sexual health programme provided the basis for translating a theory-driven approach to address our aim [11].

In line with the principles of realistic evaluation (one form of TDE), we sought evidence that would best illuminate the theory or other underlying drivers to the NCSP when it was established and how these evolved during implementation [12]. We therefore focused our data collection on sources that would enable us to track the context in which the NCSP was first considered and later delivered and to track the thinking that underpinned the programme’s initiation and implementation.

### Data collection

We constructed a timeline tracking the scientific and policy context from 1996 to 2010 using:

published research (randomised controlled trials and systematic reviews) on chlamydia screening

English Department of Health documents

NCSP strategy and annual reports

Health Protection Agency surveillance data on chlamydia diagnoses rates

To make explicit any initial theory underlying the policy to establish the NCSP and to document changes as the NCSP was implemented, we used a combination of interviews and documentary analysis:

*Interviews*: we selected a purposive sample of 14 experts, chosen because of their integral involvement in the NCSP’s establishment or implementation or because of their role as independent academic experts during the period 1996–2010. These included; national policy makers (Department of Health civil servants); people working within or advising the national team responsible for establishing the NCSP (e.g. members of the Chief Medical Officer’s Expert Advisory Group into *Chlamydia trachomatis*, NCSP Steering Group, the Independent Sexual Health Advisory Group and the National Screening Committee) and academics who have published on chlamydia control in England. The interviews, conducted by JS and PB from July 2010-Mar 2011 at UCL, in participants’ workplaces or by telephone, followed a semi-structured format (topic guide in supplementary material additional file 1), were audio-recorded and transcribed. All of the experts approached agreed to be interviewed and all were aware of PB’s current role as medical adviser to the NCSP.

*Policy documents*: to examine the stated rationale for key decisions, we searched the Department of Health online publications library using the terms “chlamydia” and “sexual health” for the period 1996–2010. This generated 209 publications of which 45 were indirectly relevant to the NCSP and 18 had direct relevance. One document (Research to inform the national media campaign teenage pregnancy in England (2000)) was not available electronically but had no apparent relevance to the programme and so was not examined.

*Commentaries*: to understand contemporary scientific and clinical opinion on chlamydia screening, we searched the Web of Knowledge database for commentaries and editorials published in peer-reviewed journals 1996–2010 using the terms “chlamydia”, “chlamydia screening”, and “NCSP”. Citation values and published responses were used as crude indicators of an article’s profile within the clinical/scientific community. We selected articles with relevance to chlamydia screening in England that were cited at least five times and/or with published responses. We identified 26 papers for inclusion into our detailed analysis.

### Analysis

We organised data from the interview transcripts, policy documents and commentaries using the Framework approach [13]. JS and PB developed a conceptual framework and independently coded several transcripts to identify themes (theories, assumptions, ideas, context) of relevance to the initiation and implementation of the NCSP. For quality assurance, ten sources were independently coded by both JS and PB, areas of discordance discussed and the coding framework refined. We coded the remaining transcripts, policy documents and articles to identify the themes emerging most prominently. We summarised all coded data sources into charts organised by theme and stage of initiation and implementation. Quotations from interviews and documents were selected that encapsulated the themes described.

### Ethical approval

We sought ethical approval for this study from UCL research ethics committee. This study was considered exempt from the requirement for ethical approval because the research involved review of publicly available information and interviews with individuals on their professional views about chlamydia screening not personal information.

## Results

### Timeline of events

**Figure 1:**
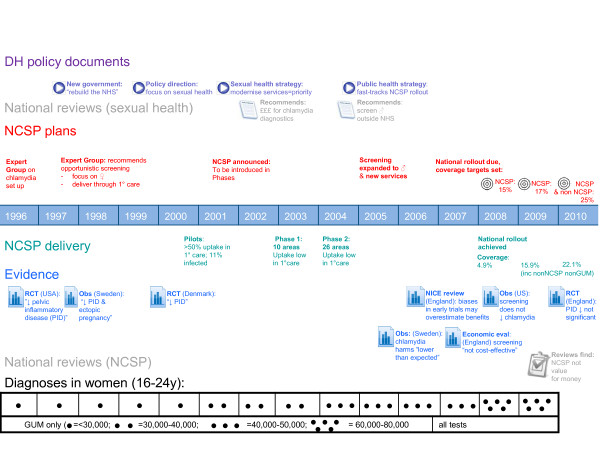
NCSP timeline 1996–2010: context, strategy and delivery.

Political and strategic developments in the NCSP are tracked against delivery and evidence for chlamydia screening (Figure 1).

#### ***The decision to introduce chlamydia screening in England (1996–2000)***

In 1996, the Chief Medical Officer (CMO) convened an Expert Advisory Group to formally consider the establishment of a chlamydia screening programme in England. The Group’s formation was in response to mounting interest in chlamydia screening in the UK [14,15], and promising observational studies of screening in other countries [16,17]. At this time, the other scientific evidence to support the effectiveness of chlamydia screening comprised one trial of screening from the USA [18]. Data from genito-urinary medicine (GUM) clinics across England showed increasing diagnoses in young people although there were no population-based estimates of chlamydia prevalence.

In 1998, the CMO published a report which proposed the introduction of chlamydia screening in England. It recommended an opportunistic screening model, targeted towards young women, delivered in general practice and community sexual and reproductive health services. It also called for a research programme including a randomised controlled trial of screening in England. The NHS Health Technology Assessment Programme subsequently commissioned cross-sectional feasibility and acceptability studies [19]. The Department of Health also funded a pilot of opportunistic screening in two areas in 1999. These pilots – where GPs were paid to offer tests - reported high uptake in primary care (>50 %) and found over 10 % of young people tested positive for chlamydia [20,21].

#### ***Establishment of the NCSP and rollout across the country (2001–2010)***

In 2001, the sexual health strategy for England 2001–2011 was published. This strategy highlighted serious problems in sexual health care, including four-week waits for urgent appointments in GUM and patchy provision outside specialist GUM services, with few GPs providing sexual health care (health promotion, advice or STI testing and treatment) other than contraception. It presented a vision of sexual health as an integrated programme, encompassing both contraception and STIs (including HIV) and proposed a new model for delivering sexual health services, where provision was expanded to primary care [22]. As part of this model, it proposed a national screening programme based on the CMO’s 1998 delivery recommendations, to be implemented in phases, with full rollout across England by 2008. Testing in GUM clinics was not part of this programme. In 2004, the Public Health White Paper accelerated the schedule, promising national provision by 2007 and announced funding to support implementation [23]. In the absence of empirical studies, modelling data estimated the level of coverage required to reduce prevalence [24]. National targets for screening coverage were first announced in 2005. From 2007, local areas were monitored against these targets.

The NCSP delivery model changed as the Programme expanded. By 2003, the target population included men. By 2004, testing was conducted outside of general practice and sexual health services in non-traditional settings such as pubs, clubs, sporting events and festivals. Implementation did not occur as planned: national provision was later than expected, numbers of people tested remained low, particularly in primary care, which accounted for less than 20 % of chlamydia test delivery, and amongst men, who account for less than 40 % of NCSP tests [25]. As a result, coverage targets were missed by a significant margin in 2007/2008. After 2008, chlamydia tests performed outside the NCSP (but not in GUM) were also counted towards coverage and targets were almost met [25].

From 2006, the validity of earlier trials was more thoroughly and widely questioned in systematic reviews, prompting more empirical research outside England to underpin screening policy [26,27]. In addition, researchers have since given greater attention to the secular changes (eg lower rates of partner change as a result of HIV prevention messages in the 1990s) that may have explained the initial fall in chlamydia diagnoses following the introduction of screening programmes [28,29]. Evidence remained equivocal: for example, in 2010 a trial in England of chlamydia screening reported a non-significant benefit of screening for reducing pelvic inflammatory disease [30]. This trial’s limitations also illustrated the significant challenges of delivering chlamydia screening even in a research context eg achieving uptake of sufficient numbers of young people [31].

### Underlying themes

#### ***Establishing the NCSP***

We did not identify a consistent theoretical basis for the NCSP. However, two underlying themes emerged that underpinned the establishment and implementation of the NCSP. We refer to the first theme as ‘explicit’ because it is central to the stated aims of the Programme and the second theme as ‘implicit’ because it was not stated in policy documents but emerged from interviews.

Theme 1: The first theme corresponds to the explicitly stated aims of the NCSP, which are to reduce chlamydia prevalence and sequelae, in response to concerns about rising diagnoses (Figure 1, dark blue boxes). Diagnosis was made possible by the advent of new technology, which was highly sensitive to detecting chlamydia and in contrast to older tests, was much less invasive, often requiring a urine test only. As the illustrative quotes below indicate, he importance of advances in diagnostic technology for the introduction of screening was corroborated by evidence from interviews, and other contemporary documents.

The CMO’s report identified chlamydia as a public health problem and screening as the policy response. This position was widely supported by the scientific and clinical community in England:

"The personal and economic costs of untreated genital chlamydial infection are considerable."

Johnson, BMJ, 1996 [15]

"The role of chlamydia in infertility is well documented: the disease may be implicated in as much as 50 % of cases."

Boag and Kelly, BMJ, 1999 [32]

"We’d been seeing chlamydia figures going up and up and there was a growing awareness that it was a major cause of pelvic inflammatory disease…. ectopic pregnancy and I suppose to my mind the trigger for all of this probably was the advent of molecular diagnostics, the idea that actually you could undertake testing using so-called non-invasive specimens."

Interview, CMO Expert Advisory Group member (1996–2001)

While the CMO’s report acknowledged that data on prevalence of chlamydia sequelae were “incomplete” and “uncertain”, its bold statements that sequelae were “severe” and that management would result in “considerable health benefit” [2] went largely unchallenged in subsequent commentaries and letters, despite the lack of trial evidence from England at that time and the incomplete knowledge concerning the prevalence and natural history of chlamydia. Instead, discussions focused on the extent to which screening would be acceptable to young people and how it should be delivered. This is illustrated in the quotes below:

Theme 2: A second theme also emerged from the interviews (Figure 1, light blue boxes). It was not referenced in policy documents, and we therefore refer to it as an implicit theme. This was concerned with the contribution of chlamydia screening to advancing wider sexual health service delivery. As described in the timeline and in interviews, sexual health services in the late 1990s/early 2000s were in urgent need of serious investment:

"The case for screening is made."

Boag and Kelly, BMJ, 1999 [32]

*"The Chief Medical Officer's plan for immediate action on* Chlamydia trachomatis*…is a step in the right direction, but it does not go far enough."*

Letter from Opaneye, BMJ, 1999 (in response to Boag & Kelly editorial) [32]

"I was shocked when I looked back at it [the CMO’s report]. And actually it doesn’t question whether there should be a screening programme, the decision has obviously been made and it’s just which target groups, which tests."

Interview, Independent academic expert (1996–2011)

"[A pilot of] opportunistic testing … achieved coverage of under 30 % among its target population…If the low response … is repeated in national pilot studies using similar methodology then few individuals are likely to achieve long term health benefits and community transmission is unlikely to be greatly reduced."

Letter from Macleod et al, BMJ, 1999 (in response to Boag and Kelly editorial) [32]

*"[The NHS pilots aimed to find out] how feasible was it to ask people to pee in a pot [*i.e. do a urine test*]… for an STI they hadn’t actually gone along to ask about in the first place."*

Interview, NCSP Steering Group member (1999–2008)

"It was all part of a growing dissatisfaction with a resurgence in STDs. There were real concerns about access to clinical services, under capacity in GUM clinics waiting times, you know that was part of the narrative that had its origins in the late 1990s."

Interview, NCSP Steering Group member (2000–2004)

Rising chlamydia diagnoses were quoted by interviewees and widely in policy documents to support claims of increasing burden of sexual ill-health:

"England is currently witnessing a rapid decline in its sexual health. Around one in ten sexually active young women (and many men) are infected with chlamydia. Syphilis rates have increased by 500 % in the last six years and those for gonorrhoea have doubled. Rates of teenage pregnancy are the highest in Europe. Sexual dysfunction is a largely silent problem within society. Sexual health services appear ill-equipped to deal with the crisis that confronts them."

Third report of session 2002–03 on sexual health, House of Commons Health Committee, 2003 [33]

As shown in the timeline and corroborated in the interview data, in contrast to the previous decade, significant efforts were now successful in gaining political recognition of this problem:

"At that point to put sexual health into a historical context, it was very much seen as the Cinderella service of all services right across the board…. We had the first ever national strategy on sexual health and that had taken 18 months or so to do."

Interview, Independent Sexual Health Advisory Group member (2002–2011)

**Figure 2:**
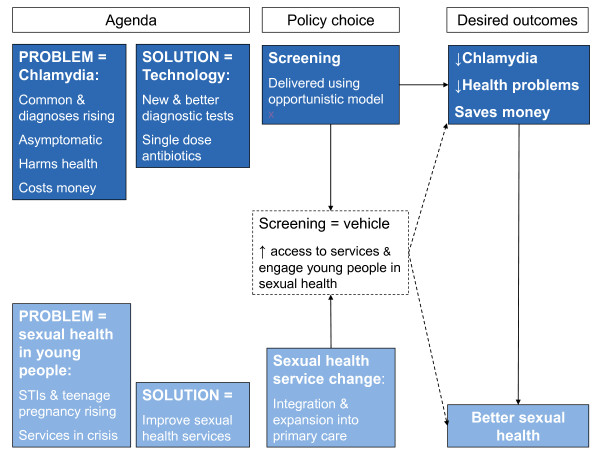
Themes underpinning the establishment and implementation of the NCSP.

Chlamydia screening was recognised as a vehicle to engage young people in discussions about their sexual health and an opportunity to drive increased access to services for management of STIs (white dotted-line box, Figure 2). This was evident from interviews and from an article by those leading the first English screening pilots:

"The proposed screening programme would demand changes in clinical practice and closer alliances between health services. This provides an opportunity for new partnerships to be formed and facilitates a more integrated approach to health care. In many ways, it heralds the approach that is required to manage the wide variety of sexual health issues that confront us today."

Pimenta et al, BMJ, 2000 [34]

"[Chlamydia screening was] an opportunity for driving up sexual health care, sexual health consultations."

Interview, CMO Advisory Expert Advisory Group member (1996–1998)

Specifically, implementing a programme of chlamydia screening was expected to expand sexual healthcare in primary care and contraceptive services:

"It was clear that what we were setting up was not just any proof of concept but a true opportunity to get STDs out of the GUM sector and into the mainstream of health protection in England."

Interview, NCSP Steering Group member (2000–2004)

"From a service delivery point of view we did also see this not only as increasing chlamydia testing and the effects on chlamydia, but also to improve access to sexual reproductive health services. If we could get more of the right people through the Screening Programme, it could have a positive effect on sexual reproductive health services."

Interview, National policy maker (2000–2005)

Although the Government frequently refers to chlamydia screening as part of its service reforms, neither expanding access to sexual health care nor engaging young people in sexual health were stated aims of the NCSP. Similarly, this theme does not feature in the CMO’s report [2], despite interview evidence that it was discussed within the Expert Advisory Group:

"[Chlamydia] was a credible relevant topic to talk about and to open up that dialogue in sexual health matters in a broader sense so there was definitely discussion about that in the CMO group."

Interview, CMO Advisory Expert Advisory Group member (1996–1998)

"What one person [in the CMO’s group] said to me was, ‘we see this Programme as being about the de-stigmatisation of sexual health services’."

Interview, National Screening Committee member (1996–2007)

The aspiration to use screening in order to expand community sexual health services and to promote discussion of sexual health with young people therefore appears as an important, but largely implicit, influencing factor in establishing the NCSP.

Our analysis also suggests however, that this implicit theme influenced key implementation decisions. These decisions included men’s eligibility for screening, where there is little empirical evidence to guide decisions; most published randomised controlled trials (RCTs) have been conducted on women [18,30]. The CMO’s Expert Advisory Group report (1998) initially recommended that screening should focus on women only. This proposal was adopted in the first phases of implementation. The CMO’s recommendation was based partly on considerations of feasibility, recognising that women are “are more likely to attend health care settings” [35]. However, the NCSP policy changed since the initial phases to recommend that programmes should screen men and women equally [36]. This was partly because studies conducted since the CMO’s report found chlamydia prevalence was similar in men and women [37,38]. However, there was still no evidence that including men in the target population for the NCSP would be cost effective in preventing chlamydia-related harms. Our interviews and documentary evidence suggest that the policy change was based less on the potential to control chlamydia, and more to promote equitable engagement of men in sexual health:

"[Screening men would] …give health professionals and researchers the opportunity systematically to investigate and address men's understanding of their sexuality and sexual behaviour."

Duncan and Hart, BMJ, 1999 [39]

"There was not robust evidence to say – when the decision was made –that screening men would be cost-effective."

Interview, NCSP Steering Group member (2000–2004)

"There was concern we were focusing chlamydia screening efforts only on women and really missing an opportunity in engaging men in sexual health."

Interview, NCSP Steering Group member (2002–2005)

"[A focus on women only] ignored the (albeit small) long-term health risks to men and, by placing the focus on women, seemed unfairly to place the entire responsibility on women too."

Men’s Health Forum, 2005 [40]

#### ***Roll out of the Programme***

The two themes evolved during implementation of the NCSP.

Theme 1: Following rollout across the country, the gaps in the evidence base to justify screening were now more widely recognised. Flaws in early RCTs and questions about the effectiveness of screening to control chlamydia and prevent reproductive ill-health led to questions about the “alacrity” with which “influential groups have adopted chlamydia screening” [41]. As the quote below shows, he gaps in the evidence surrounding the natural history of chlamydia became a central question for researchers.

"What I think we really need to know is what the natural history of chlamydia is. We just simply don’t know what we’re dealing with and on what scale and if you don’t know that you can’t know whether your benefits are going to outweigh your harms. It’s not enough to say you have some case control studies to say that pelvic inflammatory disease is associated with chlamydia or ectopic pregnancy is associated with chlamydia."

Interview, Independent academic expert (1999–2011)

These questions also led to questions about the policy of funding chlamydia screening in England:

Theme 2: The focus of the NCSP’s monitoring was entirely on delivery of testing and managing infections so any wider effects were not formally captured. Evidence from interviews suggests that early in implementation, the NCSP drove integration of services to some extent and did contribute to expanding sexual healthcare delivery beyond specialist services:

"….the Department does not know how often infection leads to serious health problems and hence whether it is cost-effective to invest so much public money in tackling this problem."

National Audit Office, 2009 [3]

"Even people who are critics of it [the NCSP] would say it’s done more to bring together, force people to talk to each other, to work together… I think without the driver of the Programme, we wouldn’t have seen it to the extent it has happened."

Interview, National policy maker (2001–2010)

There was some evidence that providers in new services used chlamydia screening as an opportunity to discuss sexual health with young people outside services.

"I do think above everything else it[offering chlamydia screening] gives the opportunity to engage in a conversation about sexual health which we’ve not been able to do before."

Interview, Local implementer (2008–2011)

As the Programme expanded, pressure to achieve high coverage led to new services focusing solely on chlamydia testing. These services became divorced from mainstream care and offered little opportunity for sexual health promotion [3]. The National Audit Office reported in 2009 that 40 % of young people tested within the NCSP by did not receive sexual health advice.

"I don’t think the intention was ever that we would set up a programme separate and different from other aspects of sexual health locally, but unfortunately that’s what seems to have evolved."

Interview, National policy maker (2001–2010)

"I’m still going to areas where they are missing a trick, that the chlamydia programme and the chlamydia staff, they’ve got a huge role to play in the teenage pregnancy agenda. It’s part of sexual health. You know, it was very much put in its own little silo and even though we wanted it to be a sustainable programme."

Interview, National policy maker (2002–2005)

"The targets take away from what we’re doing sometimes; it’s very hard for people offering screening not to feel targets are all we care about."

Interview, Local implementer (2008–2011)

In addition, our interviews reflect the conflict between achieving testing volumes and providing integrated sexual health care through chlamydia screening. The following two quotes come from two people involved during a similar period of the NCSP’s development, both working to implement the NCSP at a national level. These show that some of those involved in implementing the NCSP at a national level stated it was unacceptable for health professionals to avoid discussing sexual health with young people. However, others minimised the input required from health professionals:

"It still amazes me, last week, I was … hearing from the contraception service that … our ladies don’t come here to talk about sex and sexually transmitted infections. As far as I’m concerned that’s medically negligent."

Interview, National policy maker (2002–2005)

"The amount of time that GPs need to spend directly talking about sex with their clients is zero frankly, they may have to say have you been screened for chlamydia this year …… and if the patient said no, give them a leaflet."

Interview, National implementer Implementer (2001–2004)

## Discussion

### Key findings

We identified two concurrent themes that drove establishment of the NCSP. The first (explicitly stated in the aims of the Programme) centred on the goal to control chlamydia. The second theme (clearly articulated in interviews, but not explicitly stated in policy) was the aspiration to use chlamydia screening as a tool to achieve wider improvements in young people’s sexual health and service delivery.

### Strengths and weaknesses of the analysis

Theory-driven evaluation approaches are commonly applied for social programmes but rarely used for healthcare interventions [11]. We found that analysis of the theory underpinning a complex intervention was feasible for a public health programme and generated results directly useful to policy makers within a short time frame.

Two of the study authors (PB and JS) have been involved in chlamydia screening (PB as NCSP medical adviser and JS in evaluating the Programme) and have published on the Programme [32,42]. This position as 'insider' researchers meant that both had knowledge of and opinions on the subjects discussed in interviews. The risk of bias due to this prior involvement was minimised by explicit reference to this involvement at all stages of the analysis and input from other study authors.

This evaluation occurred several years after the establishment of the NCSP. Therefore, memories of events amongst our interviewees may be inaccurate/ incomplete. In particular, it is possible that our interviewees consciously or unconsciously adopted a revisionist position on this subject to justify what may now be perceived as an unpopular position. To address this, we tried wherever possible to seek documentary evidence to triangulate the descriptions of events in our interviews. In particular, we examined whether the implicit goal to "improve sexual health" was only a *post-hoc* justification. As evident from the illustrative quotes, the goal of improving sexual health through transforming services was referenced in documentation from 2000. Therefore, even if interviewees selectively recalled this rationale more strongly many years after the Programme was first planned, there was evidence that it existed as a rationale contemporaneously as well.

### Other related studies

The limitations in the evidence base that existed when the NCSP was established has been referred to elsewhere [4]. Our analysis builds on this literature by providing an explanation for why chlamydia screening received such widespread support despite the lack of conclusive experimental evidence underpinning the NCSP. Policy makers, clinicians and researchers recognised that chlamydia screening could be used to expand sexual health beyond specialist services and engage young people in sexual health.

The delivery of the NCSP has been subject to other critical reflection, most recently from the National Audit Office (2009) and the Parliamentary Public Accounts Committee (2010) [3,43]. These reports focused on the NCSP’s failure to reach coverage targets. They concluded that resources had been poorly used, due to “the difficulties which can arise when a national initiative is introduced into a locally-managed NHS” [3, p7]. The National Audit Office recommended that criteria for the success of the Programme should be defined. Our analysis provides an argument to consider broadening the criteria for evaluating the Programme in addition to the measures of coverage and diagnoses rates. Possibilities could include measuring the proportion of tests performed outside of specialist services and the number of young people who discussed sexual health matters during screening.

## Conclusions

Our analysis of the origins of the NCSP indicated that those involved in its establishment and implementation sought to achieve more than just chlamydia control. There were implicit aspirations to use chlamydia screening to expand sexual health services in the community and to engage young people in sexual health care. Our interviews suggest that expansion of service delivery beyond specialist GUM services has been achieved. However, this was sometimes without expected gains in service integration and did not always open a dialogue into sexual health with young people.

NHS reforms may significantly change the way in which the NCSP is delivered [44]. For example, commissioning of sexual health services by local authorities is proposed [5], and for the first time integrated data are available on chlamydia testing and positivity across all services [45]. These changes offer an opportunity for closer working between infection control and health improvement.

Having uncovered the implicit theory, future research could explicitly address it through examining the provision of sexual health advice delivered within the Programme, and by exploring the Programme’s impact on attitudes to testing and testing behaviour.

## Abbreviations

CMO = Chief Medical Officer; GP = General Practice; GUM = Genito-Urinary Medicine; NCSP = National Chlamydia Screening Programme; NHS = National Health Service; RCT = Randomised Controlled Trial.

## Competing interests

The following authors have relationships with organisations that might have an interest in the submitted work in the previous 3 years: PB and IS are employed by the HPA, which manages the delivery of the NCSP. IS was seconded to the NCSP (2006–2008); PB is medical adviser for the NCSP (2008-present). JS holds an honorary contract with the HPA and has evaluated the NCSP’s delivery. None of the authors’ spouses, partners, or children have financial relationships that may be relevant to the submitted work.

## Authors’ contributions

The initial idea for the study came from a meeting between RR, IS, GH and JS. JS and PB jointly undertook the interviews, developed the study design, conducted the analysis and interpretation of the findings. JS drafted the introduction, results and discussion sections of the paper. PB searched for policy documentation, drafted the methods section of the paper, results figures and commented extensively on each draft of the paper. GH provided guidance on study design and interim analyses and commented on all drafts of the paper. RR and IS commented on interim analyses and drafts of the paper. All authors had full access to the data used in this study and commented on all drafts of the paper and approved the final version. JS, the corresponding author had final responsibility for the decision to submit for publication. RR is the guarantor for the study. All authors read and approved the final manuscript.

## Pre-publication history

The pre-publication history for this paper can be accessed here:

http://www.biomedcentral.com/1471-2458/12/317/prepub

## Supplementary Material

Additional file 1 RATS Checklist. Click here for file
