# Supplementary material for: Chlamydia screening in England: a qualitative study of the narrative behind the policy
Source: BMC Public Health. 2012 Apr 30;12:317. doi: 10.1186/1471-2458-12-317 (PMC3458993; doi:10.1186/1471-2458-12-317)
Supplement: Additional file 1 — RATS Checklist. [file 1471-2458-12-317-S1.docx]

| **ASK THIS OF THE MANUSCRIPT** | **THIS SHOULD BE INCLUDED IN THE MANUSCRIPT** | **Reference in manuscript** |
| --- | --- | --- |
| **R Relevance of study question** |  |  |
| Is the research question interesting? | Research question explicitly state | The research question is stated in the study aims (P4) |
| Is the research question relevant to clinical practice, public health, or policy? | Research question justified and linked to the existing knowledge base (empirical research, theory, policy) | The background justifies the research question by outlining the existing knowledge base (P3) |
| **A Appropriateness of qualitative method** |  |  |
| Is qualitative methodology the best approach for the study aims?   - *Interviews:* experience, perceptions, behaviour, practice, process - *Focus groups:* group dynamics, convenience, non-sensitive topics - *Ethnography:* culture, organizational behaviour, interaction - *Textual analysis:* documents, art, representations, conversations | Study design described and justified i.e., why was a particular method (e.g., interviews) chosen? | Approach (theory driven evaluation) described and justified (p4-5) |
| **T Transparency of procedures**  *Sampling* |  |  |
| Are the participants selected the most appropriate to provide access to the type of knowledge sought by the study?  Is the sampling strategy appropriate? | Criteria for selecting the study sample justified and explained   - *theoretical:* based on preconceived or emergent theory - *purposive:* diversity of opinion - *volunteer:* feasibility, hard-to-reach groups | Purposive sample selection described and justified (p5) |
| *Recruitment* |  |  |
| Was recruitment conducted using appropriate methods? | Details of how recruitment was conducted and by whom | Sample contacted by email by either JS or PB described (p5) |
| Is the sampling strategy appropriate? |  |  |
| Could there be selection bias? | Details of who chose not to participate and why | All of the sample approached agreed to be interviewed (p5) |
| *Data collection* |  |  |
| Was collection of data systematic and comprehensive? | Method(s) outlined and examples given (e.g., interview questions) | Topic guide included (Supplementary data) |
| Are characteristics of the study group and setting clear? | Study group and setting clearly described | Interviews took place at UCL, in participants’ workplaces or by telephone (p5) |
| Why and when was data collection stopped, and is this reasonable? | End of data collection justified and described | Data collection was stopped when all the interviews were completed. |
| *Role of researchers* |  |  |
| Is the researcher(s) appropriate? How might they bias (good and bad) the conduct of the study and results? | Do the researchers occupy dual roles (clinician and researcher)? Are the ethics of this discussed? Do the researcher(s) critically examine their own influence on the formulation of the research question, data collection, and interpretation? | Researcher roles described in the methods and reflected on in discussion (p13) |
| *Ethics* |  |  |
| Was informed consent sought and granted? | Informed consent process explicitly and clearly detailed | See topic guide, supplementary material |
| Were participants’ anonymity and confidentiality ensured? | Anonymity and confidentiality discussed | All quotes were anonymised. Approval sought to acknowledge interviewees before naming them on the paper |
| Was approval from an appropriate ethics committee received? | Ethics approval cited | Statement on ethical approval (not required) included (P6-7) |
| **S Soundness of interpretive approach**  *Analysis* |  |  |
| Is the type of analysis appropriate for the type of study?   - *thematic:* exploratory, descriptive, hypothesis generating - *framework:* e.g., policy - *constant comparison/grounded theory:* theory generating, analytical   Are the interpretations clearly presented and adequately supported by the evidence? | Analytic approach described in depth and justified  *Indicators of quality:* Description of how themes were derived from the data (inductive or deductive)  Evidence of alternative explanations being sought  Analysis and presentation of negative or deviant cases | Analytical approach described on p6. Descriptive codes were first identified and analytical codes identified from these.  The coding trees are attached as supplementary material |
| Are quotes used and are these appropriate and effective? | Description of the basis on which quotes were chosen  Semi-quantification when appropriate  Illumination of context and/or meaning, richly detailed | Quotes were selected to encapsulate the theme described (p6) |
| Was trustworthiness/reliability of the data and interpretations checked? | Method of reliability check described and justified e.g., was an audit trail, triangulation, or member checking employed? Did an independent analyst review data and contest themes? How were disagreements resolved? | All participants were given an opportunity to provide feedback on the findings. 7 out of 14 did so. These participants are acknowledged with their permission in the paper |
| *Discussion and presentation* |  |  |
| Are findings sufficiently grounded in a theoretical or conceptual framework?  Is adequate account taken of previous knowledge and how the findings add? | Findings presented with reference to existing theoretical and empirical literature, and how they contribute | Findings presented following a timeline of events which details the empirical literature. |
| Are the limitations thoughtfully considered? | Strengths and limitations explicitly described and discussed | Strengths and weaknesses are covered in a specific section of the discussion (p13-14) |
| Is the manuscript well written and accessible? | Evidence of following guidelines (format, word count)  Detail of methods or additional quotes contained in appendix  Written for a health sciences audience | Topic guide and coding structures in supplementary data |
| Are red flags present? These are common features of ill-conceived or poorly executed qualitative studies, are a cause for concern, and must be viewed critically. They might be fatal flaws, or they may result from lack of detail or clarity. | *Grounded theory:* not a simple content analysis but a complex, sociological, theory generating approach  *Jargon:* descriptions that are trite, pat or jargon filled should be viewed sceptically  *Over interpretation:* interpretation must be grounded in "accounts" and semi-quantified if possible or appropriate  *Seems anecdotal, self evident:* may be a superficial analysis, not rooted in conceptual framework or linked to previous knowledge, and lacking depth  *Consent process thinly discussed:* may not have met ethics requirements  *Doctor-researcher:* consider the ethical implications for patients and the bias in data collection and interpretation | No red flags were identified by reviewers or by interviewees when findings shared for their feedback |

The RATS guidelines modified for BioMed Central are copyright Jocalyn Clark. They can be found in Clark JP: *How to peer review a qualitative manuscript*. In *Peer Review in Health Sciences*. Second edition. Edited by Godlee F, Jefferson T. London: BMJ Books; 2003:219-235
